# Hypertension in Thyroid Disorders

**DOI:** 10.3389/fendo.2019.00482

**Published:** 2019-07-17

**Authors:** Eszter Berta, Inez Lengyel, Sándor Halmi, Miklós Zrínyi, Annamária Erdei, Mariann Harangi, Dénes Páll, Endre V. Nagy, Miklós Bodor

**Affiliations:** ^1^Department of Endocrinology, Faculty of Medicine, University of Debrecen, Debrecen, Hungary; ^2^Department of Clinical Pharmacology, University of Debrecen, Debrecen, Hungary; ^3^Coordination Center for Drug Development, University of Debrecen, Debrecen, Hungary; ^4^Department of Metabolism, Faculty of Medicine, University of Debrecen, Debrecen, Hungary

**Keywords:** blood pressure, endocrine, hypertension, hypothyroidism, hyperthyroidism, arterial stiffness, cardiovascular risk, thyroid

## Abstract

Arterial hypertension represents a major global health concern; more than one fourth of the population is affected by high blood pressure. Albeit the underlying cause of the disease remains unclear in the vast majority of the cases, ~10% are of secondary origin. Endocrine disorders are common illnesses and some of them may lead to elevated blood pressure, among which thyroid diseases are of high prevalence and often overlooked, especially in mild cases. Overt and subclinical hyper- and hypothyroidism can both lead to (mostly mild) hypertension; however, the underlying mechanisms are only partially understood. The results of clinical studies are often controversial. During the past decades, some genetic mutations in the hypothalamus-pituitary-thyroid axis with cardiovascular consequences were revealed. Atherosclerotic changes resulting from lipid abnormalities due to thyroid dysfunction also affect the vasculature and can cause elevated blood pressure. The review gives a synopsis of our knowledge how thyroid hormone metabolism and functional thyroid diseases affect the cardiovascular system, their negative impact and causative role in the development of hypertension.

## Introduction

Hypertension affects 26.4% of the global adult population remaining the leading preventable risk factor for premature death and disability worldwide (1, 2). Besides the majority of patients with primary (essential) hypertension, a subgroup of ~10% of patients is affected by secondary hypertension. Among the underlying diseases several are of endocrine origin and thyroidal impairments represent an even smaller percentage of the secondary hypertension cases; their incidence and form of presentation varies with age and studied population (3). Hypertension may be the initial clinical presentation for at least 15 endocrine disorders (4), including overt and subclinical hyperthyroidism and hypothyroidism. The correction of thyroid dysfunction may normalize blood pressure (BP) in most cases, therefore checking thyroid function is essential during the workup for hypertension.

Thyroid dysfunction, both hypo- and hyperthyroidism may increase the risk of hypertension (5, 6). Hypothyroidism should be considered as a graded phenomenon with a wide variety of clinical conditions from subclinical hypothyroidism to myxedema. Subclinical hypothyroidism is a combination of serum thyrotropin (TSH) above the upper reference limit and normal free thyroxine (fT4) and free triiodothyronine (fT3) levels (7). This definition is only applicable in the absence of other acute or chronic recent or ongoing severe illness, assuming a stable thyroid function weeks or more before the evaluation and a normally functioning hypothalamic-pituitary-thyroid axis. Overt hypothyroidism is characterized by an elevated TSH, usually above 10 mIU/L, in combination with reduced circulating fT4 and fT3 levels.

While the most common cause of hypothyroidism had been environmental iodine deficiency for centuries, the situation has changed and the population became iodine sufficient or only mildly deficient. Since then, the leading causes of hypothyroidism are chronic autoimmune thyroid diseases (AITDs). Hashimoto's thyroiditis is 5–10 times more common in women than in men, characterized by an increased prevalence with age (8, 9). In AITDs the thyroid gland is infiltrated by sensitized T lymphocytes, while circulating thyroid autoantibodies can be detected as a consequence of presumably inherited defect in immune surveillance. Furthermore, hypothyroidism may occur as a consequence of radioiodine or surgical treatment for hyperthyroidism, benign nodular thyroid disease or thyroid cancer, and after external beam radiation for head and neck malignancies. Pharmacological treatment, in most of the cases administration of the iodine-containing antiarrhythmic agent amiodarone, lithium, or immune response modulators, such as interferon alfa can result in the development of thyroiditis and thyroid dysfunction; relatively new iatrogenic causes of hypothyroidism are tyrosine kinase inhibitors and PD-1 inhibitors, sunitinib, and nivolumab, respectively. Sunitinib induces hypothyroidism via reduction of glandular vascularization and induction of type 3 deiodinase enzyme (9, 10). The mechanism by which nivolumab impairs the thyroid function is not entirely understood; the explanation might be a reduction in the immune tolerance to normal thyroid tissue antigens (11). Central causes of hypothyroidism are acquired or congenital of origin; pituitary or hypothalamic tumors (including craniopharyngiomas), inflammatory (lymphocytic or granulomatous hypophysitis) or infiltrative diseases, hemorrhagic necrosis (Sheehan's syndrome), or surgical and radiation treatment for pituitary or hypothalamic disease can stay in the background of insufficient production of bioactive TSH (9).

The reported prevalence rate of overt hypothyroidism is between 0.2 and 5.3% in Europe and 0.3–3.7% in the United States, most probably due to the differences in iodine intake (12). The incidence of subclinical hypothyroidism was found to be 7.5% in the Wickham study (13), while in the Colorado study 21% among women and 16% in men, respectively (14). The levels of circulating TSH and antithyroid autoantibodies increase with advancing age; according to the NHANES III data TSH levels above 4.5 mIU/L are present in 14% of the population aged 85 and above (8).

The most common causes of hyperthyroidism are autoimmune Graves' disease and multinodular goiter. Iatrogenic hyperthyroidism can be a consequence of iodine exposure during administration of iodine-containing drugs among which far the most important is amiodarone, or excess of levothyroxine replacement therapy. Immune response modulator therapy of cancers can lead to hypo- and less frequently hyperthyroidism (15). Subacute thyroiditis is a less frequent cause of hyperthyroidism. Iatrogenic subclinical hyperthyroidism is the declared aim of T4 therapy, and is a relatively frequent condition among patients with differentiated thyroid cancer after near-total thyroidectomy. Further, a few weeks of iatrogenic overt hypothyroidism is an alternative to recombinant TSH administration before radioiodine treatment and during follow-up, in accordance with international guidelines.

The prevalence of thyrotoxicosis among women is between 0.5 and 2%, a 10-fold female predominance is present. Subclinical hyperthyroidism is a condition of mild thyroid hormone excess defined by a serum TSH concentration below the lower reference limit and normal serum fT4 and fT3 concentrations. The same biochemical pattern may describe hypothalamic or pituitary disease, non-thyroidal illness, or the pharmacologic effect of TSH secretion inhibiting drugs. The prevalence of subclinical hyperthyroidism ranges from 0.5 to 6.3%, with the highest established prevalence in individuals over 65 years; approximately half of the affected patients are on levothyroxine substitution (16).

Other relatively common thyroid diseases as multinodular goiter with euthyroidism and differentiated thyroid cancer have sparse impact on the cardiovascular system, and especially, the development of hypertension; however, a recently published meta-analysis found that hypertension significantly increases the risk of development of thyroid cancer (17).

This review focuses on the common functional thyroid disorders, hypo- and hyperthyroidism that have impact on the cardiovascular system and especially hypertension. The underlying causes of thyroid dysfunction are listed in Table 1.

**Table 1:** Causes of thyroid dysfuntions.

**Causes of hyperthyroidism**	**Causes of hypothyroidism**
**Overfunction of the thyroid**	**Congenital**
Graves' disease	Thyroid dysgenesis
Toxic adenoma	Dyshormonogenesis
Toxic multinodular goiter	Deficiency of TRH/TSH
Iodine-induced hyperthyroidism	**Acquired forms in adults**
TSH-mediated hyperthyroidism (TSH producing pituitary adenoma)	*Primary hypothyroidism*
Trophoblastic disease and germ cell tumors	Hashimoto thyroiditis
**Destructive thyroid diseases (thyroiditises)**	Iatrogenic causes:
Subacute thyroiditis	- thyroidectomy	
Hashimoto thyroiditis	- radioidine therapy	
Silent thyroiditis	- external neck irradiation	
Post-partum thyroiditis	Drugs (iodine, lithium, amiodarone, thyreostatic therapy, interferon, tyrosin kinase inhibitors, anti-CD52 monoclonal antibody etc.)
Iodine-induced thyroiditis	
**Ectopic hyperthyroidism**	Infiltrative disease
Metastatic follicular thyroid cancer	Environmental exposures
Struma ovarii	Consumptive hypothyroidism
**Exogenous hyperthyroidism**	*Central (secondary and tertiary) hypothyroidism*
“Hamburger” hyperthyroidism	*Resistance to thyroid hormone*
Overdosage of thyroxin	*Resistance to thyrotropin and thyrotropin-releasing hormone*

## Metabolism of Thyroid Hormones and Their Effects on the Cardiovascular System

Triiodothyronine is the biologically active form of thyroid hormone derived from 5′-monodeiodination of thyroxine in all tissues outside of the thyroid gland, particularly the kidney, liver, and skeletal muscle. The basal metabolic rate is affected by fT3 via altering oxygen consumption, substrate requirements and tissue thermogenesis (4). Thyroid hormones have direct and indirect cellular effects on the cardiovascular system. In hyperthyroidism systemic vascular resistance decreases as fT3 dilates resistance arterioles of the peripheral circulation, which results in the fall of the effective arterial filling followed by stimulation of renin release and activation of the angiotensin-aldosterone axis (18).

Patients with hyperthyroidism present with increased heart rate, increased pulse amplitude, and increased cardiac output by up to 300%, which resembles a state of increased adrenergic activity (6, 19), despite normal or low serum concentrations of catecholamines. Other hormonal factors are also affected: the levels of atrial natriuretic peptide, brain natriuretic peptide, endothelin-1 and the vasodilating polypeptide adrenomedullin are elevated in hyperthyroidism (6, 20). Thyroid hormone also stimulates erythropoietin secretion. Furthermore, T3 directly increases cardiac contractility, leading to widened pulse pressure (18, 20, 21). The coexistence of ischemic or hypertensive heart disease in a thyroid patient may compromise the ability of the myocardium to respond to the increased metabolic needs in hyperthyroidism and demands caution from the clinician (6).

The mortality of patients with hyperthyroidism was found to be increased by 20%, and the major causes of death are due to cardiovascular origin (22). The level of cardiac T3, as myocyte intracellular deiodinase activity is not significant, is fundamental in maintaining cardiac morphology and function in adult life via genomic and non-genomic effects. The expression of the main structural and regulatory genes is regulated by T3. Positively regulated genes as α-myosin heavy chain, sarcoplasmic reticulum Ca^2+^-ATPase, β1-adrenergic receptor, atrial natriuretic hormone and voltage-gated potassium channels also contribute to the development of cardiac output increase observed in hyperthyroidism (21). In contrary, the inhibitor of sarcoplasmic reticulum Ca^2+^-ATPase phospholamban is negatively regulated, as well as the genes of β-myosin heavy chain, adenylyl cyclase catalytic subunits, Na^+^/Ca^2+^ exchanger and thyroid hormone receptor α1 (21). Non-genomic effects of T3 can develop rapidly in the cardiovascular system not requiring thyroid hormone response element-mediated transcriptional events and altering or modulating the effects of genomic mechanisms (23).

According to earlier studies heart failure develops in 6–16% of patients with hyperthyroidism. Patients with preexisting hypertension or with risk factors for coronary artery disease have a more pronounced risk for developing hemodynamic changes leading to chronic heart failure. A relatively frequent complication of hyperthyroidism, atrial fibrillation, is an independent predictor for the development of chronic heart failure (24, 25).

Arterial stiffness is increased in hyperthyroidism (26) due to the effect of thyroxin on vascular smooth muscle and endothelial cells via genomic and non-genomic action targeting membrane ion channels and endothelial nitric oxide synthesis (27). In addition, the Calcium/ Calmodulin-dependent kinase IV (CaMKIV), which is known to be a major thyroid hormone target gene during brain development, plays an important role in blood pressure regulation through the control of endothelial nitric oxide synthase (eNOC) activity (28–30).

The hemodynamic effects of hypothyroidism are opposite to those of hyperthyroidism, although the clinical manifestations are less obvious with bradycardia being the most common sign accompanied by mild hypertension and a narrowed pulse pressure. Bradycardia, decreased ventricular filling and cardiac contractility together lead to low cardiac output. An increase in the systemic vascular resistance and slowed ventricular diastolic relaxation and filling are present. The decreased metabolic rate leads to a decline in peripheral oxygen demand, consequently heart failure is a rare clinical manifestation of hypothyroidism (31). Elevated diastolic blood pressure is present in ~30% of patients with overt hypothyroidism. Cardiac contractility and output decreases leading to a narrowed pulse pressure. In hypothyroidism renin release is decreased with an increased salt sensitivity. The consequent renal sodium reabsorption leads to an expansion of blood volume by 5.5% (5, 21, 31).

We have previously shown that in patients who had undergone thyroidectomy for differentiated thyroid cancer, increased aortic stiffness and impaired diastolic function can be detected during induced overt hypothyroidism, which is part of the diagnostic follow-up procedure (32).

Several clinical data suggest that autoimmune thyroid disease may be responsible for the development of primary pulmonary hypertension in both hyper- and hypothyroidism; therefore in pulmonary hypertension (PH) thyroid function should be evaluated (33, 34).

**Figure 1:**
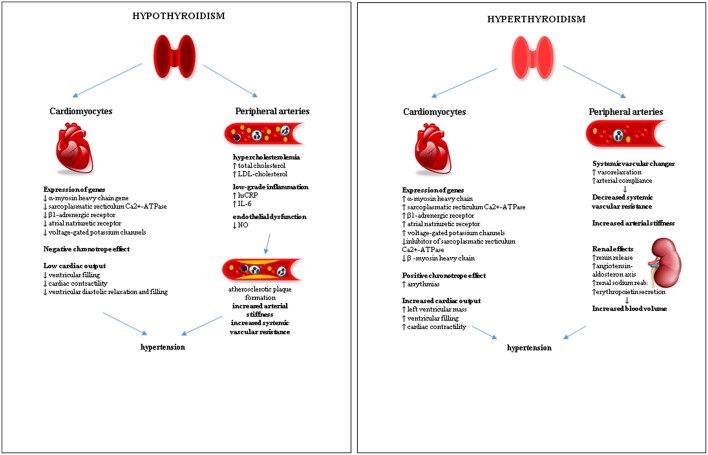
Effects of functional thyroid disorders on the cardiovascular system leading to hypertension.

The effects of the functional thyroid disorders on the cardiovascular system leading to hypertension are summarized in Figure 1.

## Genetic Background of Thyroid Function Affecting Blood Pressure

While a normal TSH and consequently fT4 and fT3 within the physiological range are essential in growth, differentiation and maintenance of adequate function of all human organs, several genetic defects have been evaluated and described in the route of thyroid hormone signaling during the past decade, including those with mutations in thyroid hormone transporters and receptors (35). According to novel studies, even minor changes in the thyroid hormone levels can affect bone mineral density (36), mental status (37), and can also lead to impaired metabolism (38), and increased cardiovascular risk (39). While the levels of the serum thyroid hormones show marked inter-individual variability, there is no significant intra-individual variability, as TSH values change very little during time (40). Based on these results a conclusion can be made that each individual has a unique thyroid set-point, defined by genetic and environmental factors such as iodine intake and smoking (41, 42).

The genetic pattern of the hypothalamic-pituitary-thyroid axis involves numerous genes with rare high-penetrance variants and common low-penetrance forms, mutations and polymorphisms, respectively. Several mutations of the hypothalamic-pituitary-thyroid axis were described to date and found responsible for impaired thyroid function (43, 44). A large number of new genes were identified during the last decade that affect the hypothalamic-pituitary-thyroid axis, some of which are responsible for thyroid dysfunction and consequently affect different organs and present risk factors for the development of different diseases, including hypertension.

Thyroid dysfunction is rarely attributed to single gene mutations. Numerous studies were performed in the last 2 years to reveal genetic variants presenting with polymorphisms associated with thyroid impairment and altered function. These encompass both linkage and candidate gene analyses targeting the hypothalamus-pituitary-thyroid pathway. A detailed overview of genes affecting the thyroid axis is found elsewhere (35). A few polymorphisms were found to be associated with blood pressure; however, the results remain controversial. A couple of type II iodothyronine deiodinase (DIO2) gene mutations were found to cause hypertension in middle-aged patients with euthyroidism (45). Interestingly, these variants did not cause any changes in the circulating TSH or free thyroid hormone levels (46, 47). These results were not confirmed by other clinical studies (48, 49). A mutation in the thyrotropin releasing hormone receptor gene was characterized to be associated with an increased risk of essential high blood pressure (50). The findings regarding gene mutations affecting blood pressure values remain controversial and need further evaluation.

## Hypertension and Increased Cardiovascular Risk in Overt Hyperthyroidism

Hyperthyroidism is accompanied by cardiovascular complications (cardiac arrhythmias, hypercoagulopathy, stroke, and pulmonary embolism) in a significant number of cases, leading to an increase in short-term morbidity and long-term morbidity and mortality. Brandt et al. found a significant 20% increase in mortality in patients with hyperthyroidism in their meta-analysis based on seven studies (22).

The excess of T3 leads to metabolic and hemodynamic changes: metabolic rate, cardiac preload, and ventricular contractility increases while systemic vascular resistance decreases, causing an increased cardiac output and hypertension, as discussed above.

The highest risk of coronary heart disease mortality and atrial fibrillation is noted when serum TSH is below 0.10 mIU/l (51).

Hemodynamic changes affect not only the left cardiac output, since pulmonary hypertension may also be present in patients with hyperthyroidism due to Graves-disease or nodular goiter (33). Pulmonary hypertension was found to be the most common complication in a study which investigated hyperthyroid patients by echocardiography (52). The prevalence of pulmonary hypertension was detected to be relatively high among hyperthyroid patients, varying between 36 and 65%, although mild and asymptomatic cases were found to be the most common (53, 54).

In the pharmacological treatment of systolic secondary hypertension caused by hyperfunction of the thyroid gland, besides reaching euthyroidism, non-selective beta-blockers are often preferred, for controlling the concomitant tachycardia and tremor effectively and blocking the peripheral T4 to T3 conversion (55).

The increased cardiovascular mortality among patients with hyperthyroidism may be as well the consequence of shared genetic or environmental factors, based on the pronounced familial aggregation of hyperthyroidism, cardiovascular disease, lifespan, and smoking habits (22).

Our group previously found that there is an increase in the aortic stiffness in combination with decreased diastolic function in patients with subclinical hyperthyroidism on levothyroxine suppression therapy after total thyroidectomy due to differentiated thyroid cancer (32).

A study found that patients with hyperthyroidism and normal blood pressure during ambulatory blood pressure monitoring (ABPM) had higher systolic blood pressure than euthyroid normotensive participants. The successful treatment of hyperthyroidism resulted in normalization of systolic blood pressure. The nocturnal decrease in blood pressure did not differ from that of normal subjects (56).

In a recently published study Lillevang-Johansen et al. investigated the association between hyperthyroidism and the occurrence of cardiovascular (CV) events among treated and untreated hyperthyroid patients (57). The real-world data of this study revealed the impact of varying thyroid status on cardiovascular events: untreated and insufficiently treated hyperthyroid patients had increased cardiovascular risk. According to the data of more than 275,000 individuals followed-up for hyperthyroidism, sufficiently controlled thyrotoxicosis did not increase cardiovascular risk. After an adjustment for main cardiovascular risk factors such as hypertension, diabetes and hyperlipidemia, the findings were not significantly affected, suggesting that elevated thyroid hormone levels are playing a major role in the increase of CV risk (57). These findings suggest that careful monitoring of treatment and maintaining euthyroidism is fundamental in the prevention of CV events among patients with hyperthyroidism.

## Hypertension and Increased Cardiovascular Risk in Subclinical Hyperthyroidism

Subclinical hyperthyroidism is defined as a subnormal serum TSH value accompanied by T4 and T3 within the normal reference range. The change in thyroid function needs to be evaluated and confirmed by a second laboratory measurement after 3–6 months (58). The prevalence of subclinical hyperthyroidism can be detected more frequently in iodine depleted areas and increases with advancing age (58). According to the Third National Health and Nutrition Examination Survey (NHANES III) 0.7% of 16,533 people were reported to have subclinical hyperthyroidism (TSH <0.1 mU/L); these subjects were not taking thyroid medication (8). Subclinical hyperthyroidism can be classified into two categories: Grade 1, with a mild decrease of serum TSH (0.1–0.4 mIU/L), and Grade 2, with a more marked TSH decrease (TSH below 0.1 mIU/L) (59).

The long exposure of the heart to subclinical hyperthyroidism leads to an altered cardiac morphology and function. As a consequence, left ventricular function changes: systolic function is enhanced, while diastolic function becomes impaired, a slowed myocardial relaxation is present resulting in an increase of left ventricular mass (60); as well as increased heart rate and arrhythmias, such as atrial fibrillation (61).

Although lipid profile is not affected unfavorably in subclinical hyperthyroidism, endothelial dysfunction and increased thrombogenicity is present (59, 62, 63).

A meta-analysis with more than 25,000 participants proved that subclinical hyperthyroidism is associated with increased risk of coronary artery disease events and coronary artery disease mortality, and also with elevated total mortality. The risk for mortality due to coronary artery disease and atrial fibrillation was more prominent in Grade 2 subclinical hyperthyroidism (51).

In the Rotterdam study involving more than 9,000 participants, the risk of cardiovascular mortality increased in a linear manner with subclinical hyperthyroidism and free T4 levels close to the upper limit of the reference range compared to those with free T4 levels close to the lower limit (64). A large systematic review with data from more than 70,000 people found that subclinical hyperthyroidism was associated with an increased risk of total mortality, heart failure, atrial fibrillation and coronary artery disease mortality in patients with Grade 2 subclinical hyperthyroidism (65).

Besides these changes in the cardiovascular risk due to subclinical hyperthyroidism, prospective cohort studies failed to show significant association between subclinical hyperthyroidism and hypertension (66, 67). Due to the increased risk for the development of cardiovascular diseases and mortality, there is a wide agreement among specialists that treatment of patients with Grade 2 subclinical hyperthyroidism should be initiated (59).

## Atherosclerosis and Hypothyroidism

Thyroid hormones affect biochemical and molecular mechanisms of lipid homeostasis resulting in a variable phenomenon of dyslipidemia (68) mostly characterized by high serum concentrations of total and low-density lipoprotein (LDL) cholesterol and normal or even elevated high-density lipoprotein (HDL) cholesterol levels. Furthermore, high serum concentrations of triglycerides, intermediate-density lipoproteins, apolipoprotein A and apolipoprotein B are frequently observed (69–71). The primary mechanism in the background of hypercholesterolemia is the accumulation of LDL cholesterol due to reduction in the number and activity of the cell-surface LDL receptors resulting in decreased hepatic LDL catabolism. In addition to the direct effect of T3 on the promoter region of the LDL receptor gene, sterol regulatory element-binding protein-2 (SERBP-2) gene is also regulated by T3, resulting in the altered transcription of 3-hydroxy-3-methylglutaryl coenzyme A (HMG-CoA) reductase and LDL receptor genes (68, 71, 72). Thyroid hormones also increase the activity of cholesteryl ester transfer protein, hepatic lipase, lipoprotein lipase, and lecithin-cholesterol acyltransferase (56, 61). Although a substantial number of studies indicated a beneficial response in patients with TSH levels between 2.5 and 4.5 concerning atherosclerosis risk factors such as atherogenic lipid parameters, impaired endothelial function, and intima media thickness (9, 73), TSH has no defined cutoff threshold regarding cardiovascular prevention and atherosclerosis (68).

A study of more than 30,000 patients showed similar results: increased LDL, non-HDL cholesterol and triglycerides and decreased HDL with high-normal TSH levels still in the reference range (74). Since the thyroid hormones have known regulatory effect on the lipid metabolism and consequently on blood pressure, their impact on the cardiovascular system may be partially explained by these mechanisms (75, 76). Another large prospective study involving more than 14,000 participants with normal TSH levels at start and followed for 11 years found that high-normal TSH levels were predictive for moderately higher blood pressure in the future. Moreover, 1 mU/L elevation in the TSH values resulted in about 2 mmHg elevation in the systolic blood pressure, 1–2 mmHg increase in the diastolic blood pressure and 0.1 mmol/L rise in non-HDL cholesterol and triglyceride values (77).

Hypothyroidism being one of the most common secondary causes of dyslipidemia (78) is clearly associated with an increased risk for atherosclerotic cardiovascular disease owing to its metabolic and hemodynamic effects (79). Atherosclerosis develops in patients with hypothyroidism as a consequence of multiple mechanisms including hyperlipidemia, hypercoagulable state, endothelial dysfunction and increased arterial stiffness which leads to arterial hypertension (80, 81).

The atherogenic lipid changes in hypothyroidism, similarly to the ones in BP values, appear to develop rapidly, as seen in individuals with a hypothyroidism only 3 weeks following thyroidectomy due to non-toxic multinodular goiter (82). Even low normal fT4 concentrations have been associated with a more atherogenic lipid profile (83). In a study involving more than 5,000 participants a significant association has been described between LDL, total cholesterol and TSH levels ranging 3.5–10 mU/L (84). Total cholesterol and LDL levels decreased in a crossover study after 100 μg levothyroxine supplementation of the subjects having TSH values greater than 6.1 mU/L (85). The same result was observed in another study conducted among individually substituted patients with TSH greater than 8 mU/L (68, 86).

Besides dyslipidemia, changes in coagulation parameters are described in thyroid disorders being partly responsible for atherogenic changes. Decreased platelet count, aggregation and agglutination, von Willebrand factor antigen and activity, decreased levels of several coagulation factors such as factor VII, VIII, IX, XI, and plasminogen activator-1 can be detected in overt hypothyroidism leading to hypercoagulability. In subclinical hypothyroidism and autoimmune thyroid disease increased fibrinogen level, factor VII level and activity, and plasminogen activator inhibitor-1 level have been detected rendering a tendency toward a hypercoagulable state (87, 88). The mechanisms underlying the development of the changes in coagulation parameters in hypothyroidism are not well understood. A possible explanation might be the direct effect of the thyroid hormones. However, it is still unclear whether the thyroid hormone deficiency or the elevated TSH levels, or both, are responsible for the alteration in the coagulation parameters (87).

Furthermore, the association between hypothyroidism and defects in the secretion of endothelium-dependent dilation factors is well known (89, 90). Endothelial cell TSH receptor induces multiple effects including the down-regulation of anti-inflammatory factors such as tumor necrosis factor-α and interleukine-6, in addition to the induction of angiogenesis (vascular endothelial growth factor, VEGF), and leukocyte adhesion (intercellular adhesion molecule-1, ICAM-1 and E-selectin). Elevated expression of leukocyte adhesion molecules is related to endothelial dysfunction, which is believed to be an early step in the development and progression of atherosclerosis. Assessment of endothelial dysfunction, as an early biomarker, is helpful in predicting cardiovascular risk and evaluating the outcome of treatment (91). Flow-mediated dilation of the brachial artery is a functional test of the capacity of increased blood flow provoked by the release of endothelial NO and other vasodilator mediators, following induced ischemia, therefore, is an accepted early method for non-invasive assessment of systemic endothelial function (92). Flow-mediated dilation was found to be impaired not only in patients with mild hypothyroidism but also in subjects with “high-normal” serum TSH levels (93). Another study also reported that patients with subclinical hypothyroidism had significantly lower flow-mediated dilation values (94).

Despite the above mentioned data, the association of subclinical hypothyroidism and atherosclerosis is still debated, although hyperlipidemia is usually present (79, 95). Carotid artery intima-media thickness measurement remains a long-standing and reliable diagnostic modality used to assess vascular morbidity at an early stage (96). A recent study revealed that carotid artery intima-media thickness is significantly higher in patients with both overt and subclinical hypothyroidism compared with normal control subjects (97).

Central arterial stiffness is positively associated with systolic hypertension, coronary artery disease, stroke, and heart failure, which are the leading causes of mortality in developed countries. Central arterial stiffening or reduced arterial compliance leads to augmented central blood pressure, increased cardiac afterload, and is an independent predictor of cardiac events (98). A previous study confirmed that hypothyroidism is associated with increased augmentation of central aortic pressure and central arterial stiffness. Furthermore, these abnormalities are reversed after adequate T4 replacement (80). Arterial stiffness was also increased in subclinical hypothyroidism and improved with L-thyroxine treatment (99).

The above detailed impairment in the lipid metabolism due to hypothyroidism may contribute to the development or progression of hypertension. In addition, patients affected by hypothyroidism may have other concomitant cardiovascular diseases and consequently their summed effect on the blood pressure may be even more accentuated.

## Hypertension in Overt Hypothyroidism

A high prevalence of diastolic hypertension had been found in patients above 50 years of age with overt hypothyroidism whose blood pressure was normalized after adequate thyroid hormone replacement therapy (100). However, hypothyroidism as a cause of hypertension is often overlooked. In an early study Saito et al. found a 3-fold higher prevalence of diastolic hypertension in hypothyroidism than in age-matched patients without thyroid disorder using the World Health Organization's (WHO) earlier used criteria for hypertension, namely blood pressure > 160/95 mmHg (100, 101). According to the current ESC guidelines for the treatment of hypertension, in-office systolic blood pressure (SBP) values ≥ 140 mmHg and/or diastolic blood pressure (DBP) values ≥ 90 mmHg define hypertension based on evidence from multiple randomized clinical trials proving that treatment of patients with these BP values is beneficial (102).

Masked hypertension, defined as a normal in-office blood pressure in spite of higher values detected during an active day, can be found during evaluation via ambulatory blood pressure monitoring (ABPM) or home blood pressure monitoring (HBPM); masked hypertension is present in ~15% of untreated patients. The prevalence of this condition is greater among younger men, who are affected by higher levels of anxiety and job stress. Furthermore, habits like smoking, alcohol consumption and more pronounced physical activity are more common among them (103). Recently published data has suggest that many individuals with masked hypertension present with a higher left ventricular mass index, therefore a higher cardiovascular risk, in the detection of which ABPM seems more efficient than HBPM (104). In a recently published pilot study, the occurrence of masked hypertension was evaluated among patients with overt and subclinical hypothyroidism using 24-h ABPM; a significantly higher prevalence of masked hypertension was found in both groups compared to controls with euthyroidism, which suggests the existence of elevated cardiovascular risk in hypothyroidism, especially in young male patients with autoimmune thyroiditis (105).

Positive association of serum TSH levels with arterial blood pressure was demonstrated through a cross-sectional analysis of pooled data from five population-based studies in adults (106). The association was present in the full range of TSH and even within the reference range, while positive correlation was limited to prevalent hypertension, and not in a 5-year change of blood pressure or incident hypertension (106). In patients who underwent thyroid surgery due to differentiated thyroid cancer and had overt hypothyroidism before radioiodine therapy, an elevation in nocturnal systolic, mean and diastolic blood pressure was found; also, an increased number of non-dippers were found among these subjects (107).

## Blood Pressure Changes in Subclinical Hypothyroidism

Hypothyroidism has been generally considered as a cardiovascular risk factor, as discussed above, stressing the need of routine screening for thyroid function especially among female patients with coronary heart disease or patients with known cardiovascular risk factors (108). Subclinical hypothyroidism is a common entity, characterized by elevated TSH-levels and fT4 and fT3 levels in the normal range.

The association between elevated blood pressure and overt thyroid disorders has been thoroughly investigated and well-established, while the question whether subclinical changes in the thyroid function present potential risk for the development and maintenance of hypertension are still under debate. Some studies detected positive correlations of subclinical hypothyroidism and hypertension in women (109, 110); however, the same results were not confirmed in men (110, 111). A number of clinical studies assessed the relationship between subclinical thyroid dysfunction, both hypo and hyperthyroidism, and endothelial dysfunction (112), arterial wall thickening (80, 99, 113), atrial fibrillation (114), and left ventricular hypertrophy (115). A study involving more than 10.000 children and adolescents found a positive correlation between elevated serum TSH levels and both systolic and diastolic blood pressure; however, this correlation was not established with hypertension (116). Upon these findings, one can presume an association between subclinical hypothyroidism and elevated risk for the development of hypertension.

In a large, population-based study with more than 30,000 participants the thyrotropin levels within the normal range were significantly correlated with the arterial blood pressure and hypertension as well in both genders; however, TSH levels above the upper limit presented positive association in women only (109). One possible explanation for this discrepancy may be that TSH levels were measured only in half of the male participants and 100% of female patients. Furthermore, these inconsistent data may also be affected by the iodine intake of the studied population; the range of TSH level may differ upon the iodine supply of the studied region (117). Studies that were conducted in regions with iodine excess like Far East Asia failed to find any correlation between blood pressure and TSH values within the normal range; these regions have higher thyrotropin reference range limits (110, 118). On the contrary, several studies performed in regions characterized by mild to moderate iodine deficiency or even sufficient iodine intake showed a positive correlation between TSH level and blood pressure values (106, 119). The differences observed in the results, however, may be partially due to the higher prevalence of hypertension in Far East Asia compared to Europe and the alternate screening and treatment methods used in these regions (120).

Overall, the data from several studies and pooled analyses suggest that the correlation between high serum thyrotropin levels and blood pressure changes might be time-dependent and cannot predict the further development of hypertension and might occur only during an actual TSH elevation (106).

In a recently published meta-analysis the effect of levothyroxine replacement therapy on blood pressure in subclinical hypothyroidism was investigated. The summed results of 10 randomized clinical trials showed that after levothyroxine therapy systolic blood pressure was reduced significantly. A subgroup analysis suggested that the systolic blood pressure lowering effect was more accentuated in patients with higher TSH levels. In 19 prospective follow-up studies both systolic and diastolic blood pressure values decreased significantly after levothyroxine initiation (121).

## Conclusions

Alterations of thyroid function may result in changes in blood pressure values as well as other traditional cardiovascular risk factors, leading to an increased cardiovascular risk, which is mild in most cases, although hyperthyroidism represents a significant elevation of cardiovascular mortality risk. The delayed clinical recognition of subclinical forms of thyroid dysfunction, i.e., subclinical hypo and hyperthyroidism has unfavorable cardiovascular effects. Available data suggest that, concerning cardiovascular risks, early diagnosis, and treatment of even mild forms of functional thyroid disorders might be beneficial in the vast majority of the patients. However, overtreatment should be avoided, and age-related or individual variances of pituitary-thyroid set-points have to be respected.

## Author Contributions

All authors listed have made a substantial, direct and intellectual contribution to the work, and approved it for publication.

### Conflict of Interest Statement

The authors declare that the research was conducted in the absence of any commercial or financial relationships that could be construed as a potential conflict of interest.
